# Heterogeneous water supply affects growth and benefits of clonal integration between co-existing invasive and native *Hydrocotyle* species

**DOI:** 10.1038/srep29420

**Published:** 2016-07-21

**Authors:** Yong-Jian Wang, Yun-Fei Bai, Shi-Qi Zeng, Bin Yao, Wen Wang, Fang-Li Luo

**Affiliations:** 1 College of Horticulture & Forestry Sciences, Huazhong Agricultural University, Wuhan 430070, China; 2 College of Life Science & Technology, Huazhong Agricultural University, Wuhan 430070, China; 3 School of Nature Conservation, Beijing Forestry University, Beijing 100083, China

## Abstract

Spatial patchiness and temporal variability in water availability are common in nature under global climate change, which can remarkably influence adaptive responses of clonal plants, i.e. clonal integration (translocating resources between connected ramets). However, little is known about the effects of spatial patchiness and temporal heterogeneity in water on growth and clonal integration between congeneric invasive and native *Hydrocotyle* species. In a greenhouse experiment, we subjected severed or no severed (intact) fragments of *Hydrocotyle vulgaris*, a highly invasive species in China, and its co-existing, native congener *H. sibthorpioides* to different spatial patchiness (homogeneous and patchy) and temporal interval (low and high interval) in water supply. Clonal integration had significant positive effects on growth of both species. In the homogeneous water conditions, clonal integration greatly improved the growth in fragments of both species under low interval in water. However, in the patchy water conditions, clonal integration significantly increased growth in both ramets and fragments of *H. vulgaris* under high interval in water. Therefore, spatial patchiness and temporal interval in water altered the effects of clonal integration of both species, especially for *H. vulgaris*. The adaptation of *H. vulgaris* might lead to invasive growth and potential spread under the global water variability.

Water availability and changes in water variability (temporal heterogeneity) are common in global environmental change^1^,^2^,^3^, such as extreme hydrological events, i.e. the distribution and duration/frequency of flooding and/or drought^4^,^5^. As status and variation of soil water resource are important to soil biota, nutrient availability and plant growth^3^,^6^,^7^, therefore, availability (distribution and amount) and temporal variability of water supply under extreme rainfall or drought may influence plant invasion^8^, further change the community dynamics^4^,^5^.

Water resource usually exhibits obviously spatial and temporal heterogeneity in natural habitats^3^,^9^,^10^. Some studies have shown that even at very small scales, e.g. the level of centimeters, can affect plant individuals^11^,^12^,^13^, i.e., two distinct types of natural wetland microhabitats (the lake shore and the nearby inland) at the centimeter level significantly affected growth of *Ranunculus reptans*^13^. Meanwhile, temporal interval of water supply such as different rainfall patterns (high and low intervals) can influence plant responses in the field^4^,^7^. A water supply with a high interval (long-time drought in-between) can be more heterogeneous than one with a low interval due to the higher temporally variability of water availability^3^. However, most studies focused on plant responses to either spatial patchiness or temporal interval^7^. Therefore, the integrative effects of spatial patchiness and temporal interval in water supply on plant responses have not been evaluated under the global rainfall change.

Clonal plants usually spread and establish with a connected-ramet system in patchy habitat with different resource supply via stolons or rhizomes^12^,^14^. The performance of these plants is often better in heterogeneous habitats by effects of physiological integration, i.e. a transport of resources from source-sites to sink-sites within the clone via stolon or rhizome connections between ramets^15^,^16^,^17^. In patchy water habitats, clonal ramets growing in high-water patches might transport water and mineral to those in low-water ones^16^,^18^. In temporal heterogeneous (high interval) water supply, the integrative ramet system can also support each other to adapt to long-time drought or flooding. However, some species showed a better performance under a more homogeneous (low interval) water supply, as these plants can take up water more stably under such conditions^7^,^10^. In results of integrative cooperative systems, plants enable buffering of any differences in water supply among ramets in fine-grained water heterogeneity and might further enhance the development of the whole plant^12^,^14^,^16^,^17^,^19^. Moreover, some studies have suggested that many of the most notorious invasive plants have the capacity for vigorous clonal propagation^19^,^20^ and their invasiveness may be closely related to clonal integration^21^,^22^. Therefore, the capacity of strong clonal integrative responses in spatial patchiness and temporal interval of water conditions may be expected to be positively correlated to species success or invasiveness. However, it is not known whether or to what extent this is the case.

According to fluctuating resource hypothesis, resources are fully utilized under non-normal conditions by either an increased supply or fluctuating, which can provide opportunity for invasion of many exotic plants^23^,^24^,^25^. When natural habitats are more or less heterogeneous, it may be more beneficial to alien plants be able to rapidly find and exploit high resource patches than native plants^1^,^2^. However, few researches have checked the cost-benefit effects of clonal integration under both spatial patchiness and temporal interval of water supply between invasive and native clonal plants. We therefore expect that successful invaders have stronger clonal integrative responses than native species. Invasiveness of clonal plants might be partly determined by their clonal growth traits (leading to being widespread and dominating a variety of habitats)^18^,^22^.

In a greenhouse experiment, *Hydrocotyle vulgaris*, a highly invasive species in China, and its co-existing, native congener *H. sibthorpioides* in wetlands were studied under spatial-temporal heterogeneity in soil water to analyze the cost-benefit effects of clonal integration. We subjected severed or no severed (intact) ramets of *H. vulgaris* and *H. sibthorpioides* to four treatments differing in spatial patchiness in water (homogeneous vs. patchy) and in temporal interval in water (low vs. high interval). The influences of clonal integration on the performance of clonal plants might vary depending on whether connected ramets experience homogeneous or patchy water conditions and on time interval in water. Therefore, we hypothesized 1) that clonal integration will improve growth of two clonal species under the heterogeneous water conditions; 2) spatial patchiness and temporal interval in water will change cost-benefit effects of clonal integration between ramets, and consequently growth of whole clonal. Base on fluctuating resource hypothesis^23^,^24^, we predicted that growth of invasive plant *H. vulgaris* may improve greater under spatial patchiness and temporal interval in water (especially patchy high interval in water supply) than the native *H. sibthorpioides* with clonal integration, and 3) *H. vulgaris* will gain greater benefits of clonal integration from spatial patchiness and temporal interval in water than *H. sibthorpioides*, especially when the resource-shortage (proximal) ramets are in low water and high interval conditions.

## Results

### Clonal growth at ramet and fragment levels

**Figure 1:**
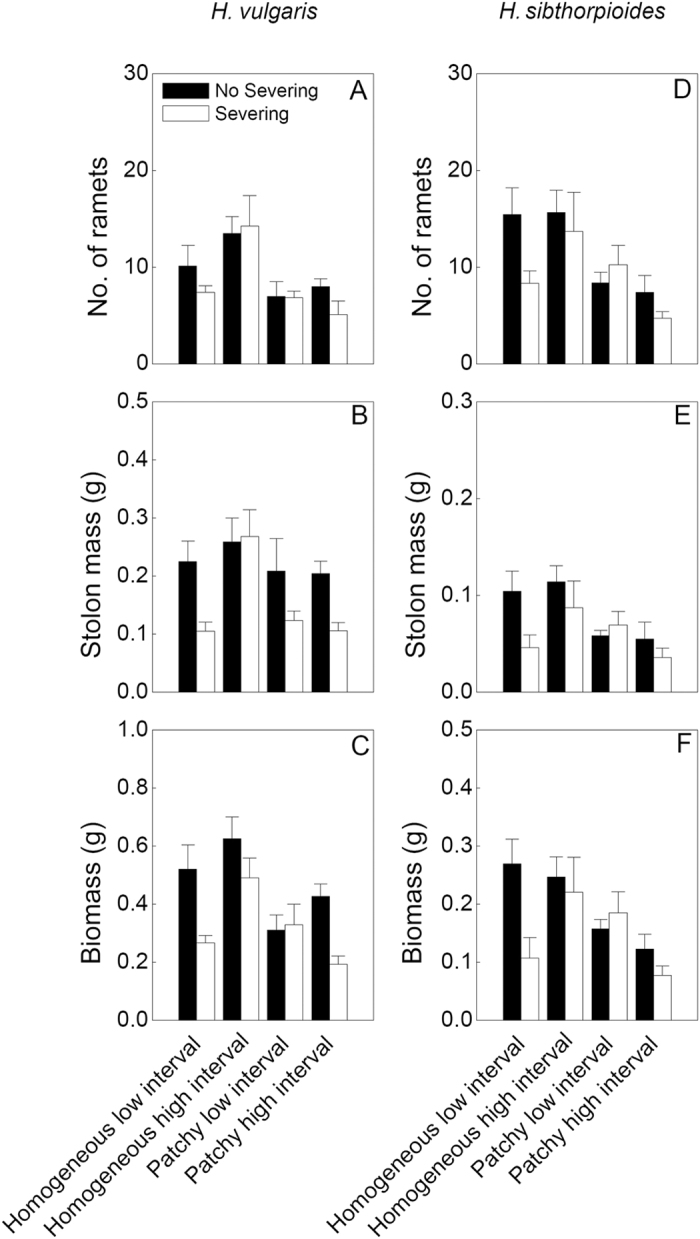
Effects of spatial patchiness (homogeneous vs. patchy), temporal interval (low vs. high interval) in water supply and stolon severing (severing vs. no severing) on number of ramets (**A**,**D**) stolon mass (**B**,**E**) and biomass (**C**,**F**) of the whole clonal fragment (proximal plus distal part) of *H. vulgaris* and *H. sibthorpioides*. Bars and vertical lines are mean values (+s.e., n = 8).

**Figure 2:**
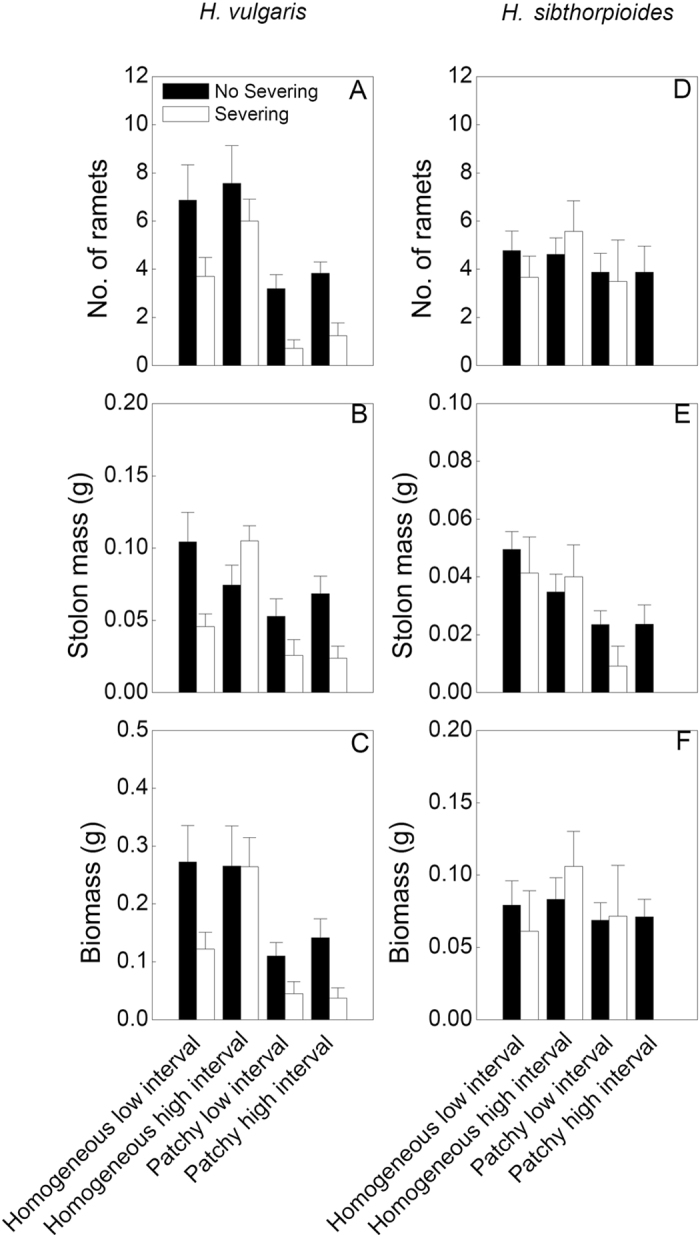
Effects of spatial patchiness (homogeneous vs. patchy), temporal interval (low vs. high interval) in water supply and stolon severing (severing vs. no severing) on number of ramets (**A**,**D**) stolon mass (**B**,**E**) and biomass (**C**,**F**) of the proximal part of *H. vulgaris* and *H. sibthorpioides*. Bars and vertical lines are mean values (+s.e., n = 8).

**Figure 3 f3:**
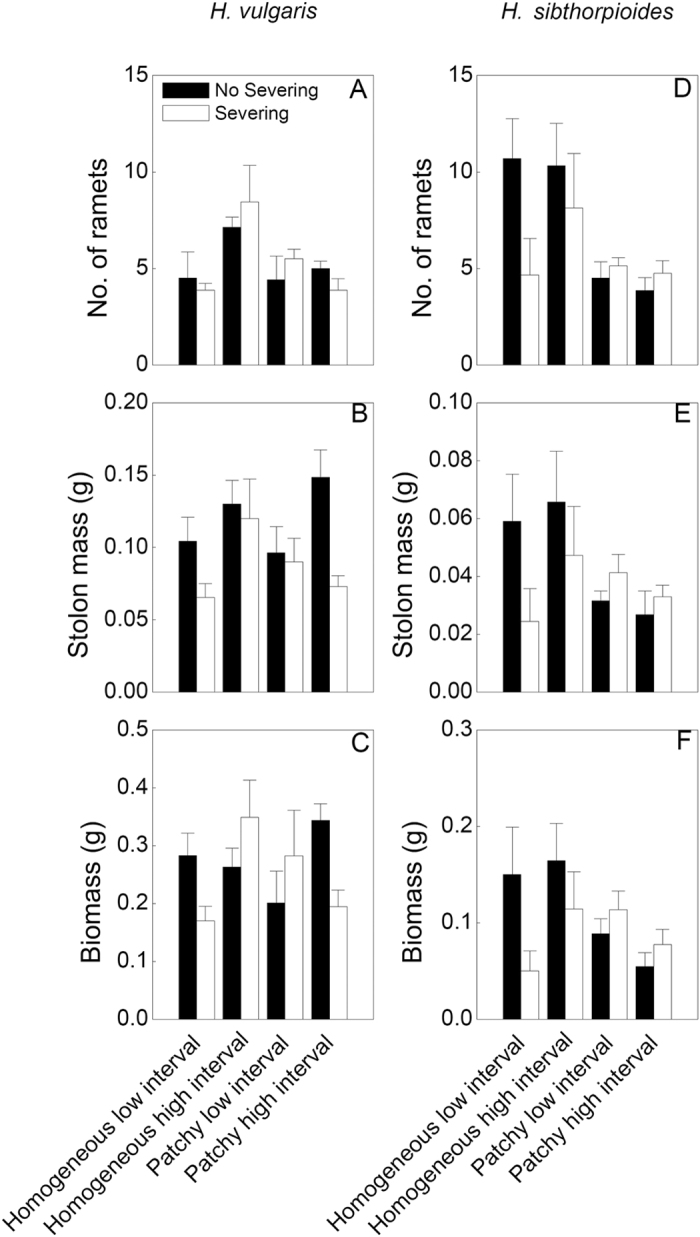
Effects of spatial patchiness (homogeneous vs. patchy), temporal interval (low vs. high interval) in water supply and stolon severing (severing vs. no severing) on number of ramets (**A**,**D**), stolon mass (**B**,**E**) and biomass (**C**,**F**) of the distal part of *H. vulgaris* and *H. sibthorpioides*. Bars and vertical lines are mean values (+s.e., n = 8).

Spatial patchiness in water supply significantly influenced the three growth measures of the ramets and the clonal fragments of both species, except for the distal part of *H. vulgaris* (Table 1). Number of ramets, stolon mass and biomass of both species were significantly greater in the homogeneous watering treatments than those in the patchy treatments (*P *< 0.05), especially in the homogeneous high interval treatment (Table 1, Figs 1,2,3).

However, the effects of temporal interval in water supply were significant for the fragments and the distal parts of *H. vulgaris* but not for these of *H. sibthorpioides*, indicating that the growth was greater in the high interval treatments compared to the low interval ones (*P* < 0.05)(Table 1, Figs 1–3). And the interactive effects of spatial patchiness and temporal interval in water supply also changed number of ramets in the distal part and the fragment of *H. vulgaris* (Table 1). Under the high interval water conditions, it produced more ramets in the homogeneous water than in the patchy water treatment (*P* < 0.05). However, under the low interval water conditions, there was no significant difference in number of ramets between two treatments of spatial patchiness (*P* > 0.05; Fig. 1). Moreover, in the patchy high interval treatment, all proximal parts of *H. sibthorpioides* were died of severing (Fig. 2D,E,F).

Clonal integration (no severing treatments) significantly improved stolon mass and biomass of these two species compared to the severing ones (*P* < 0.05) (Table 1; Figs 1–3). There were significant interaction effects of spatial patchiness in water supply, temporal interval in water and stolon severing on stolon mass and biomass (Table 1). In the homogeneous water conditions, clonal integration greatly improved the growth of the fragments of both species, and of both parts of ramets of *H. vulgaris* under the low interval but no for the high interval in water supply in water treatments (*P* < 0.05). However, in the patchy water conditions, the significant effects of clonal integration significantly increased the growth in both ramets and fragments of *H. vulgaris* (*P* < 0.05) but no for *H. sibthorpioides* (*P* > 0.05) under the high interval in water treatment (Table 1; Figs 1–3). These results suggest that the spatial patchiness and temporal interval in water partly altered the effects of clonal integration of both species, especially for *H. vulgaris*.

Compared to the native species *H. sibthorpioides*, the invasive species *H. vulgaris* accumulated higher biomass and stolon mass in all treatments (*P* < 0.05) (Figs 1–3).

### Cost-benefit analysis

**Figure 4:**
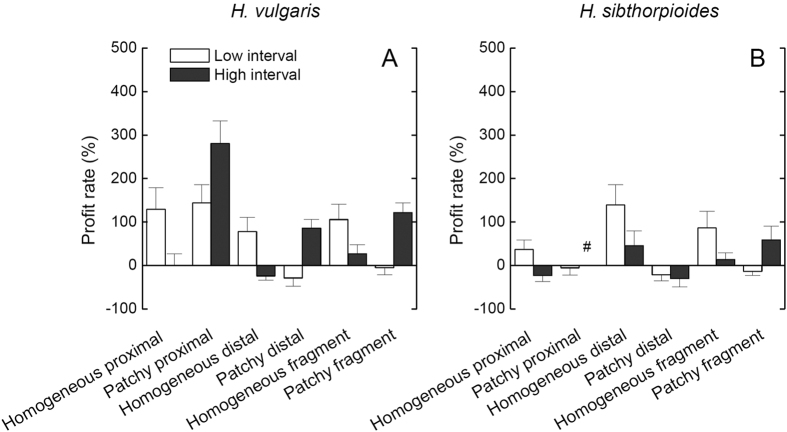
Cost-benefit analyses between spatial patchiness (homogeneous vs. patchy), temporal interval (low vs. high interval) in water supply between *H. vulgaris* (**A**) and *H. sibthorpioides* (**B**) at the fragment and the patch levels. ^#^represents that there is no value of profit rate in the patchy proximal ramets under the high interval water conditions according to the formula, but the real profit rate is great, due to the high difference in biomass between no severing (0.071 ± 0.012) and severing (0) treatments. Bars are mean values (+s.e., n = 8).

The profits of clonal integration were higher in *H. vulgaris* than in *H. sibthorpioides*, in the resource-shortage (proximal) ramets than in the relative resource-rich (distal) ramets under the patchy, high and low [Fig f3]interval treatments (Fig. 4A,B). In consistent with the results of growth measures, the homogeneous low interval and the patchy high interval treatments can greatly increase the benefits of clonal integration in both parts of ramets and fragments of *H. vulgaris*. Therefore, *H. vulgaris* can get more profits in the homogeneous low interval and the patchy high interval treatments. However, *H. sibthorpioides* mainly get more profits in the homogeneous low interval watering conditions.

## Discussion

Although many studies have tested effects of spatial patchiness or temporal heterogeneity in the supply of water on growth or clonal integration of plants^3^,^7^,^10^,^12^,^14^,^16^, none has examined the effects of both spatial and temporal heterogeneity in water supply. Our results obviously indicated that both spatial patchiness and temporal interval in water supply could partly alter growth and clonal integration of both species, especially for the invasive species *H. vulgaris*. Clonal integration improved the growth of both species, i.e. producing higher number of ramets, stolon mass and biomass, suggesting that clonal integration is very important for both species in habitats of water variability.

In the homogeneous water conditions, clonal integration greatly improved the growth in the fragments of both species, as well as both parts of ramets of *H. vulgaris* under the low interval in water supply. Previous studies have indicated that sharing of resources between connected ramets could increase the performance of clonal plants when ramets experience not just contrasting levels of resource availabilities in heterogeneous environments^12^,^14^,^16^, but also the different stages of resource availabilities in homogeneous environments^26^,^27^. It has been generally accepted that ramets of clonal fragments are often in different developmental stages and differ in resource uptake ability^28^,^29^, and experience the different levels and temporal variability of external resource supply^26^,^27^,^30^. Then, in the relatively homogeneous conditions, the effects of clonal integration on plant performance may depend on resource uptake ability and external resource supply^27^. The homogeneous low interval in water supply was a relatively steady (relative soil moisture (RSM) with low temporal variability between 32–46%) and resource supply was relatively high (RSM was higher than 32%) compared to the high interval one. Thus, resources might be easily transported from proximal ramets to distal ramets and integration could increase plant performance resulted from proximal ramets. The proximal ramets might take up higher amounts of resources than distal ramets when exposed to such relatively high resource conditions (the homogeneous low interval in water)^27^. In the field, many natural habitats and some anthropogenic habitats are probably relatively homogeneous in space^29^ and heterogeneous in time^7^,^10^, such as some shallow wetlands where water movement can homogenize fine-scale conditions. In consistent with a recently study in such relatively homogeneous water supply, the net effects of integration on clonal performance should be more positive at the high levels of spatially uniform water supply^26^,^27^. The findings further supported the source-sink hypothesis, suggesting that differences in resource uptake drive the sharing process, with resources moving from ramets with high uptake ability or favorable resource to resources to those with low uptake ability or unfavorable resource with carbohydrates, water and mineral by clonal integration.

However, in the patchy water conditions, clonal integration significantly increased the growth in both parts of ramets and fragments of *H. vulgaris*, but not for *H. sibthorpioides*, under the high interval in water. In the patchy high interval treatment, resource might be transported from (distal) ramets in relatively high water (RSM was about 50%) to (proximal) ramets in low water (RSM was about 10–20%) when fragment grew in the period of continuing watering, partly driving by relative resource-sink in water. When the fragments grew in the period of no watering, both ramets might share resource from (distal) ramets in the moderate water supply (RSM was about 30%) and (proximal) ramets in the extremely low water supply (RSM was less than 10%) or support each other due to the high fluctuating water conditions. Furthermore, growth and reproduction of clonal fragments depend not just on resource sharing but also on other aspects of clonal integration, e.g. sharing the risk^26^,^30^. Another possible reason for the shifting effects of integration is that the proximal ramets of *H. vulgaris* might possess higher uptake ability than the distal ramets at the beginning of the establishment, while the situation was reversed after the establishment of young ramets. That is why the resource sharing and the benefits of integration between the intact ramets were bidirectional in *H. vulgaris*.

The performance of *H. vulgaris* and *H. sibthorpioides* was better in the homogeneous water than in the heterogeneous water conditions, which is consistent with the previous studies^7^,^10^,^28^,^31^. Plants can take up water more stably and have a better performance under more homogeneous conditions of water supply^3^,^28^. Especially in the homogeneous high interval in water supply, the ramets and the clonal fragments accumulated higher biomass in the homogeneous high interval in water supply regardless of with or without stolon severing. The resource transportation may not occur when resource gradient is not large enough and resource is rich for growth of both ramets^29^,^32^. Besides, the high interval (water variability) in water supply might inhibit the integration in the uniform water conditions.

The source-sink driving force between the intact ramets of clonal fragments can be affected by the differences both in resource uptake ability and in resource availability^28^. The benefits of clonal integration between the intact ramets were bidirectional for *H. vulgaris* but unidirectional for *H. sibthorpioides*. *H. vulgaris* gained greater benefits of clonal integration from the spatial patchiness and the temporal interval in water supply than *H. sibthorpioides*, especially for the proximal ramets distributed in the unfavorable patches. Similarly, a higher clonal growth of *H. vulgaris* fragments was achieved in the previous studies in the heterogeneous environment^30^,^33^,^34^. Another possible explanation for different effects of the spatial patchiness and the temporal interval in water supply on performance of these two species might be that the plasticity in some of their clonal growth traits associated with adaptability to varied water conditions differed greatly^31^,^35^. Previous studies have indicated some associations between the degree of plasticity in some of key growth traits of clonal plants and plant adaptability to changing environmental conditions^16^,^36^. The high plasticity of key physiological and morphological traits is usually interpreted as an adaptive response to environmental variability^17^, especially for invasive plants^16^,^17^,^30^. The physiological and morphological traits may also vary in the entire clones or the clonal fragments, especially by clonal integration in a temporally unstable environment. For instance, through clonal integration, physiological plasticity allows ramets rapidly promote water uptake capacity of roots in response to unpredictable water supply especially in habitats with inherently droughty soils, or plant leaves can immediately increase their photosynthetic capacity when exposed to sunflecks interrupting periods of low light^17^,^32^. Furthermore, in consistent with the hypothesis that *H. vulgaris* accumulated great growth in the high interval (variability) water conditions, physiological and morphological plasticity plays a significant role in adaptation of the temporal interval in water supply. Thus, the different benefits of integration between these two species can reflect the different performance of their clone fragments in the spatial patchiness and the temporal interval in water supply.

To our knowledge, this is the first study to compare the interactive effects of spatial patchiness and the temporal heterogeneity in water supply, and clonal integration on growth between invasive and native *Hydrocotyle* species. The results showed the significant interactive effects on the growth and the benefits of integration of both species, especially for the invasive species *H. vulgaris*. The better performance of *H. vulgaris* might lead to invasive growth and potential widespread under the water variability. Nowadays, severe climatic events attributable to climate change have already had serious consequences for invasion of clonal plants^2^,^8^,^20^,^22^. In our further studies, multispecies comparison of invasive and native clonal plants in the interaction between the spatial and the temporal heterogeneity of water supply under global climate change needs to be considered.

## Methods

### Ethics statement

The experimental plants were obtained from the wetlands, so no permission was requested for the collection. The experiment was conducted in a greenhouse that was established by the research team, and thus no special permission was requested for the experiment. The experiment did not involve any endangered or protected species.

### The species

*Hydrocotyle vulgaris* L. (Apiaceae), a perennial clonal herb originating from Europe and North America, is commonly distributed in a broad range of habitats, from semi-moist to wet conditions in bogs, valleys and dune grassland^30^,^33^,^37^. It can form large clones by producing stolons^34^. Each node along the stolons has the capacity of forming a ramet that consists of one petiolate leaf and adventitious roots^33^. *H. vulgaris* produces flowers and fruits from March to September in the field, but the dispersal mainly relies on clonal propagation via stolon fragments rather than sexual reproduction^34^. The species was introduced in China as a garden species in the 1990 s and recently the coverage enlarges. It has already escaped from the aquarium trade and expanded widely into native plant communities by vegetative propagation and been considered significantly invasive in China^33^,^34^.

*Hydrocotyle sibthorpioides* Lam. (Apiaceae) is a stoloniferous perennial clonal species that is native to Asia^38^. It can adapt to a wide variety of conditions, ranging from relative dryness to complete submergence, in habitats as diverse as forest understorey, mountain slopes, and grasslands to wet valleys and wetlands^39^. This species produces stolons with rooted ramets on its nodes. Interconnected ramets via stolons often locate in heterogeneous water environment from wet conditions to dry grasslands. Although *H. sibthorpioides* flowers from April to June and produces plenty of seeds, this species mainly relies on clonal propagation via stolon fragments for spreading.

*H. vulgaris and H. sibthorpioides* are co-existed species in the wetland habitats, and not endangered or protected species, and no specific permissions are required for this location to collect plants. Both species produce slender stolons along which rooted ramets are formed. In the field, most of stolons are distributed on the ground or in the shallow soil (less than 1 cm deep). The distance between adjacent connected ramets (spacer length) varies between 1 to 4 cm. The density of ramets can be higher than 50 dm^−2^. Plants of *H. vulgaris and H. sibthorpioides* used in this experiment were collected from three locations in the wetlands of Yezhi Lake (30°28′13′′N; 114°19′34′′E) and Jiefang Park (30°36′39′′N; 114°17′31′′E) in Wuhan, Hubei Province, China. The minimal distance between sampled locations of each wetlands is at least 200 m to expect that ramets from different locations belong to different genotypes^30^. These ramets were mixed and propagated vegetatively in a greenhouse at Forestry Experiment Center of Huazhong Agricultural University. Ramet pairs, each consisting of two new (first-year) ramets connected by a stolon, were cut off and randomly assigned to the experimental treatments. Ramet pairs of each species were uniform in size. One ramet in each pair was recognized as the initial proximal part, indicating its relative proximity to the mother stolons, while the other as the initial distal part^17^.

### Experimental design

A total of 64 ramet pairs were transplanted into plastic pots with a divider (diameter = 18 cm, height = 18 cm) for each species filled with 2.2 L of a even mix of sand and yellow-brown soil (1:1 v:v) with 5 g of slow release fertilizer (Osmocote, N–P–K: 15–9–12, releasing for 5–6 month). The tested ramet pairs used in the experiment were similar-sized (5.13 ± 0.42 cm in length, 0.028 ± 0.006 g in dry mass for *H. vulgaris*; 4.64 ± 0.37 cm in length, 0.021 ± 0.004 g in dry mass for *H. sibthorpioides*; means ± SE), and no differences between treatments were detected in initial size of this plants (*P* > 0.05 for both species, independent *t* test).

The proximal and distal ramets of each ramet pair were planted in two equal parts (patches) of a pot. A physical barrier (18 cm long × 11 cm deep) was built in-between two patches of each pot. The barriers were 11 cm high and sealed all in the soil (11 cm deep) to prevent water penetration between patches. A 2 × 2 cm slice was made on the barrier so that the stolons connecting the two ramets could pass through easily. The ramet pairs were allowed to recover for two weeks before the start of the experiment.

**Figure 5:**
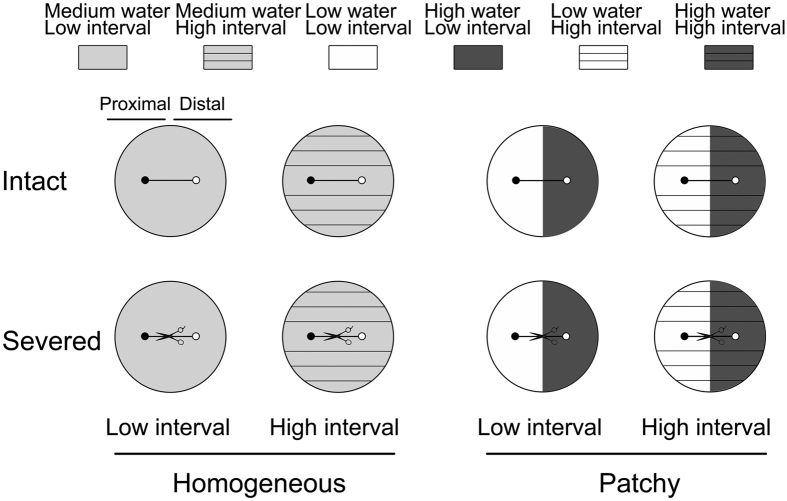
Experimental design. The experiment used a factorial design with two stolon severing treatments (intact vs. severed), two treatments of temporal interval in water (low vs. high interval) and two treatments of spatial patchiness in water (homogeneous vs. patchy). In the low interval treatment, tap water was added daily for the first three days, and without watering in the following three days; in the high interval treatment, tap water was added daily for the first 15 days, and without watering in the following 15 days. In total, there were 10 and 2 watering cycles respectively in the low and the high interval treatment during the experiment. In the homogeneous treatment, both proximal and distal ramets of a pair were subjected to the medium water conditions (80 mL water per day); in the patchy treatment, the proximal ramet of a pair was subjected to the low-water conditions (5 mL water per day), while the distal ramet was subjected to the high-water conditions (75 mL water per day).

The experiment used a three-way factorial randomized block design with eight replications (Fig. 5). The factors were stolon severing with two levels (severing or no severing, i.e. without or with clonal integration), spatial patchiness in water with two levels (homogeneous, 80 mL of water per day for whole pot, and patchy, 75 mL or 5 mL of water per day in the high-water or the low-water patch, respectively), and temporal interval in water (water supply frequency) with two levels (low interval, cycle of 3-d watering and 3-d no watering, and high interval, cycle of 15-d watering and 15-d no watering). The stolon connecting the two ramets of a pair was cut off in the stolon severing treatment and left untreated in the intact (no severing) treatment.

Therefore, there were four types of spatial patchiness and temporal interval in water, in the homogeneous low interval treatment, a watering cycle was that 80 mL tap water was evenly added to the whole pot per day for the first 3 d, and then without watering in the following 3 d. In the homogeneous high interval treatment, the cycle was adding 80 mL tap water evenly in the whole pot per day for 15 d, and then without watering in the following 15 d. In patchy low interval treatment, the cycle was adding 75 mL tap water in the high-water patch and 5 mL tap water in the low-water one per day for 3 d, and then without watering 3 d. In patchy high interval treatment, the cycle was adding 75 mL tap water in the high-water patch and 5 mL tap water in the low-water one per day for 15 d, and then without watering for the following 15 d. The experiment was conducted for 60 days, so there were 10 watering cycles for low interval treatments, and two cycles for high interval treatments. In the patch watering treatment, the proximal ramet of a pair was subjected to the low-water treatment, while the distal ramet was subjected to the high-water treatment. The water volume for each watering was supplied not to exceed the capacity of soil absorption. The total volume of water supplied during the treatment was the same among all different treatments. The treatments that combined different spatial patchiness in water supply with different watering frequencies started at the third week after the transplantation. The pots were randomly assigned to different positions on a bench in a plastic film greenhouse.

The experiment was conducted from October to December 2014 (60 days) in the greenhouse under natural sunlight in the forestry experimental center of Huazhong Agricultural University. During the experiment, the mean temperature and relative humidity in the greenhouse were about 18.2 °C and 65.0%, respectively (measured by Amprobe TR300, Amprobe, Everett, WA, USA). The light intensity in the greenhouse was about 80% of the light intensity at the outside. Soil moisture (water content by volume) was monitored with a soil moisture meter TZS-II (HEB Biotechnology Co., Xi’an, China) in the selected four replications of each watering treatment combination during the experimental period. Measurements were taken daily before the watering. Relative soil moisture content (RSM) was calculated as the difference between the measured value and the minimum value during the experimental period, divided by the range between the maximum and minimum values during the experimental period.

### Measurements and data analysis

Before harvest, we counted the number of ramets in the proximal and the distal parts separately. Then, ramets in each part were separated into petioles, leaf blades, stolons and roots, dried at 80 °C for 48 h and weighed. Total biomass was the sum of dry mass of petioles, leaf blades, stolons and roots. The biomass, stolon mass and number of ramets of a clonal fragment were the sum of the proximal and the distal parts.

We used three-way ANOVAs to test effects of spatial patchiness (homogeneous vs. patchy), and temporal interval (low vs. high interval) in water supply, stolon severing (severing vs. no severing) and their interactions on number of ramets, stolon mass and biomass of the clonal fragment, the proximal part and the distal part. If significant effects were detected, then *Tukey* multiple comparison tests were used to compare the means between the treatments. Costs and benefits of clonal integration were defined as the difference in performance of the proximal and distal parts between severed and no severed ramets, which were calculated separately for the proximal and distal parts in terms of biomass.

The profit rate was calculated as^17^: *PR* = (*B*_*ns*_−*B*_*s*_)/*B*_*s*_. Where *PR* is the profit rate of ramets or fragment of each species, *B*_*s*_ is the mean value of biomass of severed ramets or fragment across the eight replicates, and *B*_*ns*_ is the value of biomass of no severing ramets or fragment in each replicate. Values of PR are symmetrical around zero. Positive values indicate facilitation, while negative values indicate obstruction and zero indicates neutral. *T*-test was conducted to analyze the differences of profit rates between treatments. All statistical analyses were carried out with SPSS 18.0 (SPSS, Chicago, IL, USA). Prior to ANOVAs, all data were checked for normality and homoscedasticity. The differences were considered to be significant if *P* < 0.05.

## Additional Information

**How to cite this article**: Wang, Y.-J. *et al*. Heterogeneous water supply affects growth and benefits of clonal integration between co-existing invasive and native *Hydrocotyle* species. *Sci. Rep*. **6**, 29420; doi: 10.1038/srep29420 (2016).

## Figures and Tables

**Table 1:** Results of three-way ANOVAs for effects of spatial patchiness (homogeneous vs. patchy), temporal interval (low vs. high) in water supply and stolon severing (severing vs. no severing) and their interactions on growth of the whole clonal fragment (A), the proximal part (B) and the distal part (C) in *Hydrocotyle vulgaris* and *Hydrocotyle sibthorpioides*.

	Patchiness (P)	Interval (I)	Severing (S)	P × I	P × S	I × S	P × I × S
*Hydrocotyle vulgaris*							
(a) Fragment							
No. of ramets	15.53***	6.80*	0.38	7.80**	0.08	0.06	2.10
Stolon mass	6.26*	5.72*	6.26*	3.57	0.53	0.82	13.90***
Biomass	8.12**	3.88*	7.20**	2.37	0.59	0.34	6.28*
(b) Proximal							
No. of ramets	23.71***	1.79	9.88**	0.34	0.01	0.23	0.29
Stolon mass	17.58***	1.29	6.94**	0.18	1.33	3.58	7.92**
Biomass	17.37***	0.75	5.56*	0.30	0.01	0.90	5.04*
(c) Distal							
No. of ramets	3.35	4.84*	0.03	8.85**	0.04	0.03	2.10
Stolon mass	0.06	4.51*	6.73*	0.87	0.01	0.87	7.27**
Biomass	0.08	4.19*	4.40*	1.66	0.73	0.63	5.61*
*Hydrocotyle sibthorpioides*							
(a) Fragment							
No. of ramets	8.64**	0.01	1.69	2.50	1.18	0.01	1.63
Stolon mass	4.28*	0.16	4.30*	1.76	0.64	0.23	2.40
Biomass	8.45**	0.57	4.36*	2.88	3.19	0.70	4.40*
(b) Proximal							
No. of ramets	5.43*	0.30	1.94	2.76	1.67	0.21	3.07
Stolon mass	9.14**	0.99	6.83*	0.82	3.83	0.01	3.04
Biomass	4.02*	0.11	1.96	3.36	0.79	0.05	1.30
(c) Distal							
No. of ramets	7.56**	0.14	1.39	0.54	2.96	0.52	0.40
Stolon mass	4.33*	0.06	2.15	0.46	3.52	0.35	1.14
Biomass	4.34*	0.21	3.67	1.12	7.05*	1.10	5.11*

The given are *F* values and significance levels (***P < 0.001, **P < 0.01, *P < 0.05). Degree of freedoms were 1, 64 for all the effects.
